# Analysis of Thermoelastic Contact of Gas-Lubricated Rough Sealing Faces

**DOI:** 10.3390/ma17143600

**Published:** 2024-07-21

**Authors:** Shaoxian Bai, Yangyang Chen, Jing Yang

**Affiliations:** College of Mechanical Engineering, Zhejiang University of Technology, Hangzhou 310032, China; yangyang19970228@163.com (Y.C.); yangjing@zjut.edu.cn (J.Y.)

**Keywords:** surface roughness, thermoelastic contact, gas lubrication, face seals

## Abstract

Friction and wear are the main failure sources of face seals. When the surfaces of sealing rings exhibit greater roughness, the level of friction might increase and lead to sealing failure. Therefore, in this paper, based on the elastic contact hypothesis of rough and wavy surfaces and the influence of temperature on the elastic modulus of materials, a thermoelastic contact lubrication model of a gas-lubricated end seal is established. The novelty and advantage of this study is that it takes the effect of surface roughness into consideration during thermoelastic analysis of gas-lubricated seals. The film pressure, temperature, contact force and deformation of a gas spiral groove-faced seal are numerically determined. The influence of surface roughness on the contact distribution, deformation and temperature of the end-face seal at different speeds and pressures is analyzed. The film thickness increases as the rotational speed increases from 1 rpm to 2000 rpm, while the contact pressure sharply decreases from 0.25 kPa to 0. The analysis shows that the roughness contact mainly happens on the inner side of the rings due to convergent distortion of the seal faces, which easily causes partial wear of the seal faces. Moreover, it can also be found that the spiral grooves on the sealing surface can produce obvious hydrodynamic pressure effect due to the function of shear speed when the speed increases to 2000 rpm, while the film temperature increases from 293.3 K to about 306 K. The greater surface roughness results in a larger temperature rise under low-rotational-speed and lower-seal-pressure conditions, which further increases the risk of severe wear or even failure of the seal faces.

## 1. Introduction

Contact between rough surfaces is the direct cause of friction and the main cause of wear failure of face seals [1,2]. With the continuous application of gas face seals in aero-power machinery, the problem of thermoelastic contact has become an increasingly restrictive factor for the long life and reliable design of seals in the face of complex and variable conditions such as frequent start–stop, high temperature and high speed.

At present, the mixed-lubrication model based on Greenwood–Tripp (GT) model [3] and Patir–Cheng (PC) model [4,5] is widely used in the thermal effect analysis of the mixed lubrication of the seal face, and the two rough surfaces of the friction pair are equivalent to a smooth rigid surface and a rough elastic surface. The GT model is close to the actual engineering situation and lays a foundation for research on contact problems in contemporary Tribology. In 1978, Pair and Cheng [4,5] proposed an average flow model (PC model for short) considering the contact conditions of micro-surface roughness peaks, and a calculation method for the influence of surface roughness on the flow of lubricating fluid was given by introducing the flow factor into the Reynolds equation. In 1997, Ruan [6] analyzed the mixed lubrication characteristics of mechanical end seals based on the mixed lubrication model established by combining the PC model and the GT model.

With the application of groove technology in face sealing, the superposition of surface roughness and geometric groove on the face of the seal has further affected the bearing capacity of the sealing fluid. In a previous study, Lebeck carried out systematic theoretical and experimental research on the circumferential surface waviness in liquid lubricated contact mechanical seals, focusing on their thermoelastic instability [7], hydrodynamic lubrication [8], two-dimensional model [9] and waviness effect [10], showing that the circumferential waviness of the carbon ring surface after operation can be generated with an amplitude between 0.5 and 0.8 μm, resulting in an obvious hydrodynamic pressure effect. For the aqueous medium under pressure of 2.87 MPa and 93 °C, when the wave number is 3 and the dimensionless wave amplitude increases from 1 to 5, the proportion of liquid film bearing capacity can increase to 95%. When the wave number is nine, the hydrodynamic pressure effect is enhanced, and the proportion of liquid film bearing capacity can be increased to 100%, which can significantly reduce the contact wear.

Surface contact friction heating forms a temperature gradient in the radial direction of the seal ring, and the uneven distribution of thermal deformation on the seal face directly affects the contact state of the seal face, which changes from the theoretical surface contact to linear contact, resulting in poor fitting of the end face, an increased leakage rate and radial contact bias wear. In 1976, Li’s research pointed out that the thermal deformation of the liquid contact mechanical seal would cause the deformation of the seal end face in the amplitude of 2 μm, resulting in the convergence of the clearance and resulting in the contact wear of the seal ring at the inner diameter [11]. In 1986, Doust and Parmar were the first researchers to experimentally measure the thermal deformation of the non-contact wedge seal, which was in good agreement with the theoretical results calculated by the boundary integral equation method [12]. In 2015, Blasiak’s research on heat transfer and thermal deformation in a non-contact liquid end seal showed that when the rotating speed was 800 rad/s, the temperature of the sealing fluid lubrication film could rise by 16 K from the outer diameter to the inner diameter, resulting in the convergence and deformation of 0.7 μm seal clearance [13].

For gas seals, the thermal deformation is also prominent (as observed in radial tapered face seals studied by Thomas in 2007 [14] and grooved faced seals by Xie in 2020 [15] and Zhu in 2021 [16,17]). Xie’s work [15] shows that with the increase in ambient temperature, the thermal deformation reduces the opening force slightly, while the thermal viscosity effect significantly reduces the leakage rate. When the ambient temperature is increased from 300 K to 700 K, the opening force and leakage rate are reduced by 3.7% and 76%, respectively. The temperature difference between inside and outside diameters has a greater influence on the thermal deformation of the sealing surface. With the decrease in opening force and the increase in the leakage rate, the sealing surface will have a sharp divergence deformation. From 0 to 75 K, with the increase in temperature difference, the opening force decreases by 12% and the leakage rate increases by 224%. Theoretically, the thermal deformation problem not only leads to the complexity of the contact distribution, but also the temperature change leads to a change in the contact force. However, the effect of rough contact on seal properties such as temperature rise is not clearly quantified, especially considering coupling factors such as deformation of seal face.

In this paper, based on the elastic contact hypothesis of rough and wavy surfaces and the influence of temperature on the elastic modulus of materials, a thermoelastic contact lubrication model of a gas-lubricated end seal is established. The gas film pressure, temperature distribution, contact force and deformation of the spiral groove in the end face seal are numerically solved. The influence of surface roughness on the contact distribution, deformation and temperature of the seal end face at different speeds and pressures is analyzed.

## 2. Model Development

**Figure 1:**
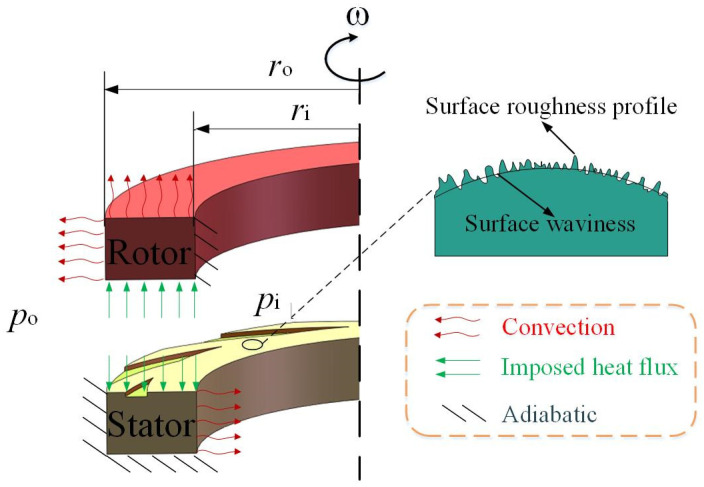
Schematic of spiral face gas seal and thermal boundary conditions.

Figure 1 illustrates a typical gas spiral face seal, which consists of a smooth ring and a spiral-grooved one. In the analysis, a pressure–periodic boundary condition is applied, so a periodic computing region is defined by the dashed lines, as illustrated in the figure.

### 2.1. Thermoelastic Contact of Rough Surfaces

**Figure 2:**
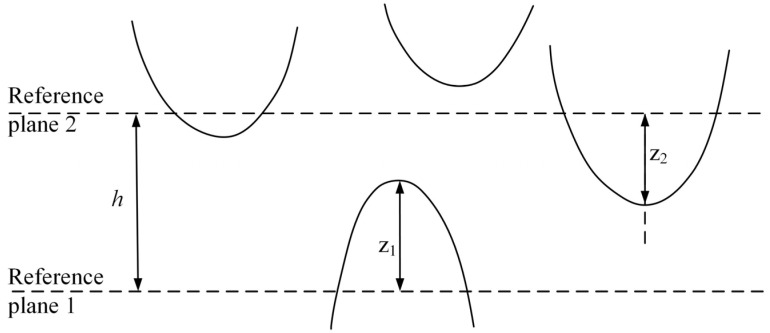
GT contact model [3].

Here, we describe the method used to create the one-dimensional topography parameters, contour arithmetic mean deviation or center line mean *R*a, shown in Figure 2. It is the arithmetic average of the height of each point on the contour within the measured length range [18]:

(1) $$Ra=\frac{1}{L}\int_{0}^{L}\left|z(x)\right|dx$$

where *z*(*x*) is the contour height of each point and *L* is the measured length.

The contour root-mean-square deviation or root-mean-square value *σ* is defined as [18]

(2) $$\sigma =\sqrt{\frac{1}{L}{\int_{0}^{L}z\left(x\right)}^{2}dx}$$

For a rough surface with Gaussian distribution, the relationship between the *R*a value and *σ* is [18]:

(3) $$Ra=\sqrt{\frac{2}{\pi }}\sigma =0.798\sigma$$

*σ*′ is the standard deviation of the combined roughness of two surfaces of *σ*_1_ and *σ*_2_ [18].

(4) $$\sigma^{\prime }=\sqrt{\sigma_{1}^{2}+\sigma_{2}^{2}}$$

*E*′ is the plane-stress modulus for the material, calculated from the elastic modulus *E*_1_, *E*_2_ and Poisson ratio *υ*_1_, *υ*_2_ of two rings [18].

(5) $$\frac{1}{E^{\prime }}=\frac{1}{2}\left(\frac{1-v_{1}^{2}}{E_{1}}+\frac{1-v_{2}^{2}}{E_{2}}\right)$$

The influence of temperature, *T,* on the elastic modulus, *E,* can be expressed as follows [18]:

(6) $$E=E_{0}e^{-\alpha T}$$

Here, for the austenitic stainless steel, *E*_0_ = 31.673 and *α* = 3.9929 × 10^−4^.

When the mechanical seal is running, the heat has two main sources: the shear action of the fluid and the contact action of the solid. The frictional heat generated by surface contact is introduced into the energy equation of the gas film as the gas film heat source term.

According to the GT contact model, the nominal pressure caused by rough peak contact is obtained [3]:

(7) $$p_{t}={\left[1+\alpha \left(T-T_{0}\right)\right]}^{2}\frac{16\sqrt{2}}{15}\pi {\left(\mu \beta \sigma \right)}^{2}\sqrt{\frac{\sigma }{\beta }}E^{\prime }F_{2.5}\left(\lambda \right)$$

where *µβσ* is the roughness characterization parameter, between 0.04~0.08 [19,20]; *λ* is the ratio of film thickness to roughness, *λ* = *h*/*σ*.

Assuming that the height of the surface roughness peak conforms to the Gaussian distribution, the calculation formula of *F*_2.5_(*λ*) is as follows [3]:

(8) $$F_{2.5}(\lambda )=\frac{1}{\sqrt{2\pi }}\int_{\lambda }^{\infty }{\left(s-\lambda \right)}^{2.5}e^{-\frac{s^{2}}{2}}ds$$

The actual contact area is as follows [1]:

(9) $$A\left(\lambda \right)=\pi^{2}{\left(\mu \beta \sigma \right)}^{2}\tilde{A}F_{2}\left(\lambda \right)$$

where $\tilde{A}$ is the nominal contact area and the calculation formula of *F*_2_(*λ*) is as follows [3]:

(10) $$F_{2}(\lambda )=\frac{1}{\sqrt{2\pi }}\int_{\lambda }^{\infty }{\left(s-\lambda \right)}^{2}e^{-\frac{s^{2}}{2}}ds$$

According to the furrow effect [21], the relationship between the cutting force *F*_e_ and the actual contact area is calculated as follows.

(11) $$F_{e}=A\sigma_{s}$$

where *σ*_s_ is the yield limit of the material, *σ*_s_ = 206.8 MPa in the flowing analysis. Here, the cutting force is often considered as the friction force.

So, the friction coefficient *f* can be calculated as follows:

(12) $$f=\frac{A(d)\sigma_{s}}{\tilde{p}(d)}=\frac{\pi^{2}{\left(\mu \beta \sigma \right)}^{2}\tilde{A}F_{2}\left(\frac{d}{\sigma }\right)\sigma_{s}}{\left(\frac{16\sqrt{2}}{15}\right)\pi {\left(\mu \beta \sigma \right)}^{2}E^{\prime }\sqrt{\frac{\sigma }{\beta }}\tilde{A}F_{2.5}\left(\frac{d}{\sigma }\right)}=\frac{15\pi F_{2}\left(\lambda \right)\sigma_{s}}{16\sqrt{2}E^{\prime }\sqrt{\frac{\sigma }{\beta }}F_{2.5}\left(\lambda \right)}$$

Further, according to the frictional heat formula, the heat generated by solid contact is obtained:

(13) $$w_{\mathrm{fric}}=fp_{t}\omega r$$

### 2.2. Governing Equations

Since the magnitude of the film thickness is close to the magnitude of the roughness peak, the influence of the surface roughness on the flow of the medium must be considered. The average Reynolds equation with a contact coefficient and flow direction coefficient is introduced [22]:

(14) $$\frac{\partial }{r\partial \theta }\left(Q_{\theta }\frac{\rho h^{3}}{\eta }\frac{\partial p}{r\partial \theta }\right)+\frac{\partial }{\partial r}\left(Q_{r}\frac{\rho h^{3}r}{\eta }\frac{\partial p}{\partial r}\right)=6\omega Q_{s}\frac{\partial \rho h}{\partial \theta }$$

where *Q*_θ_ is the circumferential pressure flow factor, *Q*_r_ is radial pressure flow factor, and *Q*_s_ is shear flow factor.

(15) $$\left\{\begin{array}{ll} Q_{\theta }=1-Ce^{-\gamma (\frac{h}{\sigma })} & \gamma \leq 1\\ Q_{\theta }=1+C{\left(\frac{h}{\sigma }\right)}^{-\gamma } & \gamma >1 \end{array}\right.$$

(16) $$Q_{r}\left(\frac{h}{\sigma },\gamma \right)=Q_{r}\left(\frac{h}{\sigma },\frac{1}{\gamma }\right)$$

(17) $$\left\{\begin{array}{ll} Q_{s}=A_{1}{\left(\frac{h}{\sigma }\right)}^{\alpha_{1}}e^{-\alpha_{2}\left(\frac{h}{\sigma }\right)+\alpha_{3}{\left(\frac{h}{\sigma }\right)}^{2}} & h/\sigma \leq 5\\ Q_{s}=A_{2}e^{-0.25\left(\frac{h}{\sigma }\right)} & h/\sigma >5 \end{array}\right.$$

where *γ* represents the directionality of asperity. *A*_1_, *A*_2_, *C*, *α*_1_, *α*_2_ and *α*_3_ are the calculation parameters [4,5].

The clearance between two rings can be given as follows:

(18) h=h0+hwavecos(Nwθ)Non−groove areah0+hg+hwavecos(Nwθ)Groovearea

where *h*_0_ is the minimum film clearance and *h*_g_ is the groove depth; *h*_wave_ is the wave amplitude and *N*_w_ is the number of waves.

By adding the friction heat to the energy equation of the gas, we can obtain the following equation [22]:

(19) $$\begin{array}{l} q_{\theta }\frac{\partial T}{r\partial \theta }+q_{r}\frac{\partial T}{\partial r}=\frac{\eta \omega^{2}r^{2}}{h\rho c_{v}}-\frac{h^{3}}{12\eta \rho c_{v}}\left[Q_{\theta }{\left(\frac{\partial p}{r\partial \theta }\right)}^{2}+Q_{r}{\left(\frac{\partial p}{\partial r}\right)}^{2}\right]\\ +\frac{fp_{t}\omega r}{\rho c_{v}}+\frac{k_{g,s1}}{\rho c_{v}}\left(T_{s1}-T\right)+\frac{k_{g,s2}}{\rho c_{v}}\left(T_{s2}-T\right)+R_{u}\rho \frac{\partial Th}{\partial t} \end{array}$$

where

(20) $$\begin{array}{l} q_{\theta }=-Q_{\theta }\frac{h^{3}}{12\eta }\frac{\partial p}{r\partial \theta }+Q_{s}\frac{\omega rh}{2}\\ q_{r}=-Q_{r}\frac{h^{3}}{12\eta }\frac{\partial p}{\partial r} \end{array}$$

where *c*_v_ is the constant specific heat capacity of gas; *k*_gs1_ and *k*_gs2_ is the convection heat transfer coefficient between gas and ring; *T*_s1_ and *T*_s2_ is the ring’s surface temperature.

For the stator ring, the heat conduction equation is given as follows [22]:

(21) $$\frac{\partial^{2}T_{s1}}{r^{2}\partial \theta^{2}}+\frac{\partial }{r\partial r}\left(r\frac{\partial T_{s1}}{\partial r}\right)+\frac{\partial^{2}T_{s1}}{\partial z^{2}}=0$$

Since there is motion in the direction of *θ*, the rotor ring’s heat conduction equation is obtained as shown in [22]

(22) $$\frac{\partial^{2}T_{s2}}{r^{2}\partial \theta^{2}}+\frac{\partial }{r\partial r}\left(r\frac{\partial T_{s2}}{\partial r}\right)+\frac{\partial^{2}T_{s2}}{\partial z^{2}}=\omega \frac{\rho_{s2}c_{s2}}{k_{c2}}\frac{\partial T_{s2}}{\partial \theta }$$

where *k*_c2_ is thermal conductivity of the rotor ring; *ρ*_s2_ is the density of the rotor ring; *c*_s2_ is the specific heat capacity of rotor ring.

Here, assuming the gas molecules are rigid spheres, according to the principle of equal energy portions, the energy per degree of motion freedom is equal to *E*m. Further, we assume that the gas temperature only represents the macroscopic inner energy of the gas molecular, so the following equation can be obtained [22].

(23) $$T=\frac{i_{d}E_{m}}{c_{v}}$$

where *i*_d_ is the freedom number of gas motions and *c*_v_ is the specific heat at a constant volume. Here, *i*_d_ = 5 for the oxygen gas.

Further, it is assumed that gas pressure is determined only by both translation energy of gas molecules and gas density. Hence, the pressure component induced by the gas can be expressed as follows [22]:

(24) $$p=\frac{R_{u}\rho i_{d}E_{m}}{c_{v}}$$

where *R*_u_ is the universal ideal gas constant and the value is 8.31434 J/(mol·K).

### 2.3. Boundary Conditions of the Fluid Film

Equation (1) can be solved for the density and pressure distribution of the gas film. In the analysis, pressure–periodic boundary condition is applied as follows [22]:

(25) p(r,θ=π/N)=p(r,θ=−π/N)

Equation (2) can be solved for the temperature distribution of the gas film. The dynamic temperature boundary condition is applied as follows [22]:

(26a) $$T(r=r_{o},\theta )=T_{\mathrm{inlet}}\;\mathrm{if}\;q_{r}(r=r_{o},\theta )<0$$

(26b) $$T(r=r_{i},\theta )=T_{i}\;\mathrm{if}\;q_{r}(r=r_{i},\theta )>0$$

where

(27) $$q_{r}=-\frac{h^{3}}{12\eta }\frac{\partial p}{\partial r}$$

$q_{r}(r=r_{o},\theta )<0$ and $q_{r}(r=r_{i},\theta )>0$ mean that the gas flows from ambient into the lubricating region at the boundaries.

In addition, temperature–periodic boundary condition is expressed as follows [22]:

(28) T(r,θ=π/N)=T(r,θ=−π/N)

For a compressible fluid, the exit pressure is not equal to the surrounding inner pressure when the flow is choked [23,24]. The exit condition is expressed as follows [22]:

(29) $$p_{\mathrm{exit}}=\frac{M_{1}}{M_{\mathrm{exit}}}\sqrt{\frac{2+(1+\gamma_{1})M_{1}^{2}}{2+(1+\gamma_{1})M_{\mathrm{exit}}^{2}}}p_{1}$$

where *M*_exit_ is the Mach number at the seal exit with gas pressure *p*_exit_ and density *ρ*_exit_, and *M*_1_ is the Mach number at any point near the exit with gas pressure *p*_1_ and density *ρ*_1_. Then, the expression of parameter *γ*_1_ can also be obtained [22].

(30) $$\gamma_{1}=\frac{\ln\left(\frac{p_{1}}{p_{\mathrm{exit}}}\right)}{\ln\left(\frac{\rho_{1}}{\rho_{\mathrm{exit}}}\right)}$$

At the inlet of the seal, pressure and temperature losses can be expressed as follows [22]:

(31) $$p_{\mathrm{inlet}}=\frac{p_{o}}{{\left[1+\frac{(\gamma_{2}-1)M_{\mathrm{inlet}}^{2}}{2C_{L}^{2}}\right]}^{\frac{\gamma_{2}}{\gamma_{2}-1}}}$$

(32) $$T_{\mathrm{inlet}}=\frac{T_{o}}{1+\frac{(\gamma_{2}-1)M_{\mathrm{inlet}}^{2}}{2C_{L}^{2}}}$$

where the loss coefficient *C*_L_ is equal to 0.65 [16]. *M*_inlet_ is the Mach number at the seal inlet with gas pressure *p*_inlet_ and density *ρ*_inlet_. Then, the expression of parameter *γ*_2_ can be obtained [22].

(33) $$\gamma_{2}=\frac{\ln\left(\frac{p_{\mathrm{inlet}}}{p_{2}}\right)}{\ln\left(\frac{\rho_{\mathrm{inlet}}}{\rho_{2}}\right)}$$

where *p*_2_ and *ρ*_2_ are gas pressure and density, respectively, at any point near the inlet.

### 2.4. Boundary Conditions Relating to the Solids

Here, convective, adiabatic and imposed heat boundary conditions are used on surfaces as shown in Figure 1, and the heat exchange condition between solid and ambient is as follows [22]:

(34a) $$-k_{c1}{\left(\frac{\partial T_{s}}{\partial n}\right)}_{s}=k_{\mathrm{gs}1}(T_{s1}-T_{0})$$

(34b) $$-k_{c2}{\left(\frac{\partial T_{s}}{\partial n}\right)}_{s}=k_{\mathrm{gs}2}(T_{s2}-T_{0})$$

**Figure 3:**
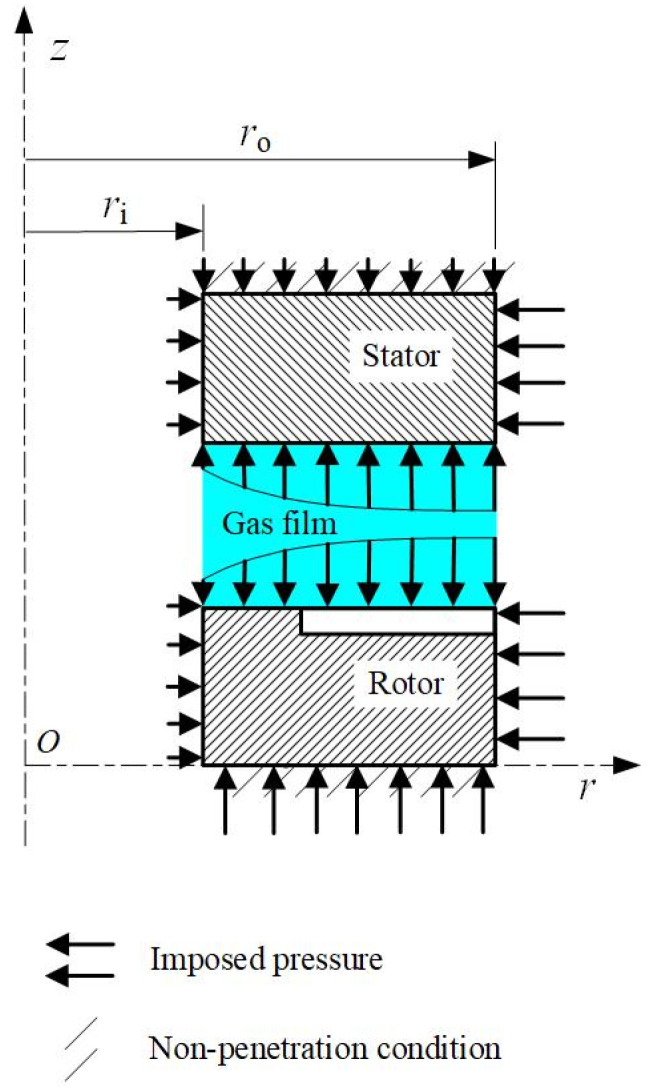
Mechanical boundary conditions.

Figure 3 presents the geometry of the solids and the mechanical boundary conditions. The fluid film between the faces is delimited by inner and outer radii (*r*_i_ and *r*_o_). On the rear face of the stator (the rotor), the area bounded by the inner radius (the balance radius) and the outer radius, *r*_i_ and *r*_o_, respectively (*r*_b_ and *r*_o_, respectively), can be in contact with the ring support. Meanwhile, the O-ring location on the stator, *h*_oring_, is also of concern, as it determines the influence of sealed pressure acting on the outside cylindrical surface of the stator. Non-penetration conditions with a rigid surface are imposed on rear surfaces of the stator and the rotor, with the ring supports being assumed to be rigid.

### 2.5. Numerical Method

The finite difference method is utilized to obtain the film pressure, film temperature and ring temperature. The finite element method is used for the coupling calculation of the face elastic and thermal distortions.

In numerical analysis, the opening force is expressed as follows:

(35) $$F_{\mathrm{open}}=\int_{0}^{2\pi }\int_{0.5d_{i}}^{0.5d_{o}}prdrd\theta$$

The closing force can be calculated as follows:

(36) $$F_{\mathrm{close}}=\pi \left(r_{o}^{2}-r_{b}^{2}\right)p_{o}+\pi \left(r_{b}^{2}-r_{i}^{2}\right)p_{i}$$

The solid contact force can be calculated as follows:

(37) $$F_{\mathrm{cont}}=\int_{0}^{2\pi }\int_{r_{i}}^{r_{o}}p_{t}rdrd\theta$$

Average gas temperature, *T*_av_, is defined as follows [22]:

(38) $$T_{\mathrm{av}}=\frac{\iint Trd\theta dr}{\iint rd\theta dr}$$

The convergence criterion is defined as follows [22]:

(39) $$\delta X=\left|\frac{X^{j}-X^{j/2}}{X^{j}}\right|$$

where $X=\left\{p,T,T_{s},T_{\mathrm{av}}\right\}$ and *j* is the iterative number.

**Figure 4:**
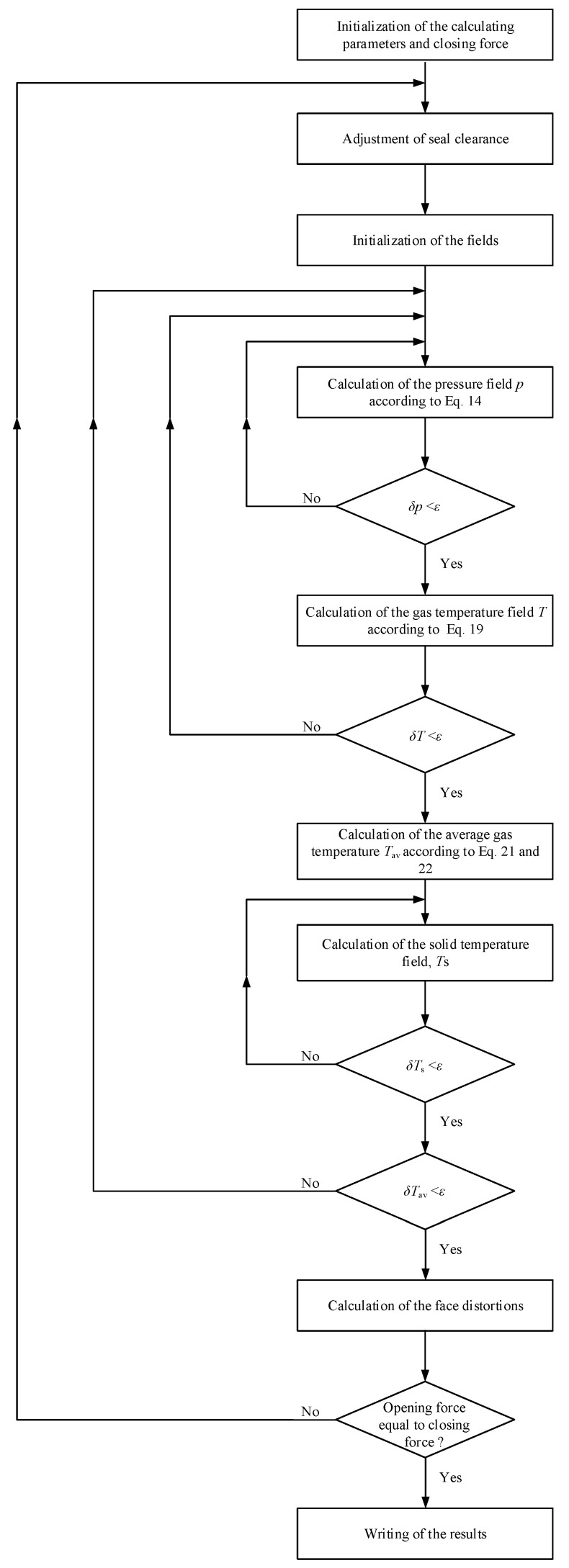
Algorithm of the program.

The mesh density of gas film is 80 × 80, and that of rings is 80 × 80 × 30. The value of error limit, *ε*, for the convergence criterion is 10^−5^. The film pressure, film temperature, ring temperature, face distortions and seal clearance are successively calculated into four overlapping loops as shown in Figure 4. The new equilibrium position of the seal clearance is then established, and the entire iterative process is repeated until the convergence criterion on the equilibrium position is satisfied.

In the following numerical analysis, the dimensions of the seal, the parameters of asperity and the sealing materials are shown in Table 1, Table 2 and Table 3 respectively. The proposed model can not only apply for graphite and steel, but also for other sealing pairs if the material parameters are changed.

## 3. Result and Discussion

For the gas face seal, the rough contact directly dependents on clearance between two sealing faces, which mainly affected by operating parameters and surface roughness. In the following analysis, we put points on the influence of rotational speed, seal pressure and surface roughness on the frictional performance.

### 3.1. Lubrication Status Transition with Rotational Speed

Generally, lubrication status transits from mixed lubrication to full-film lubrication with the increase in rotational speed. Meanwhile, the film thickness increases, which leads to a sharp decrease in contact pressure. In theory, the face deformation affects the surface contact distribution and changes with the sealing speed and film thickness. In this section, we calculate the influence of rough surface contact on lubrication state after coupling face deformation.

**Figure 5:**
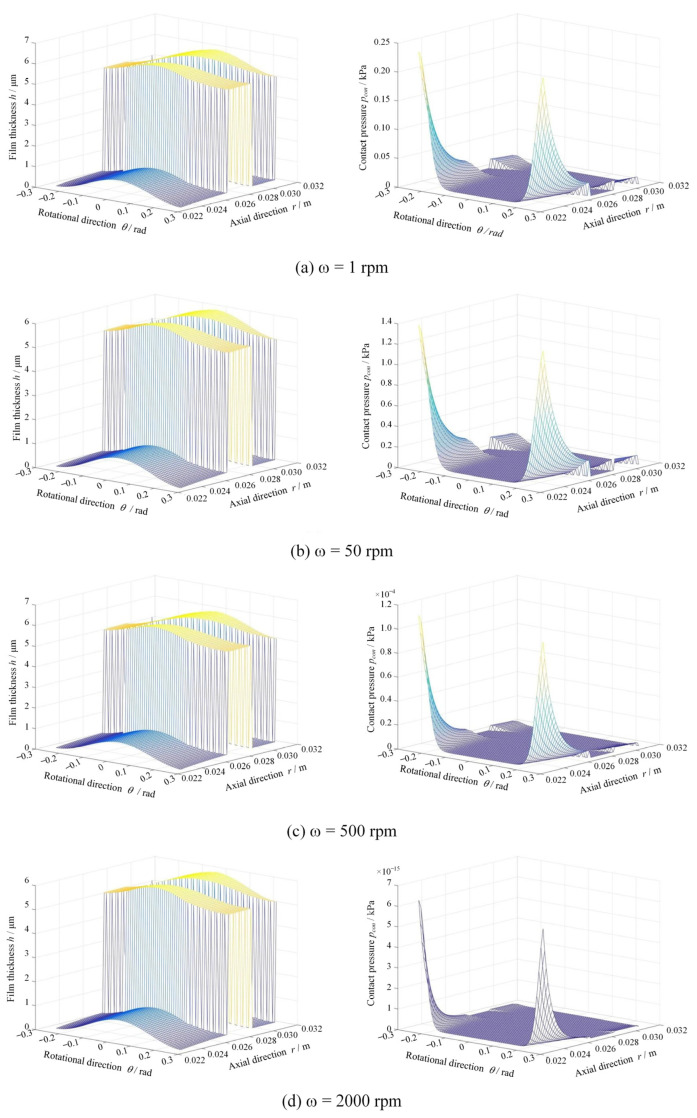
Film thickness and contact pressure distributions under different rotational speed (*R*a = 0.20 μm, *p*_o_ = 1.1 MPa).

Figure 5 presents the film thickness and contact pressure distributions under different rotational speeds at *R*a = 0.20 μm and *p*_o_ = 1.1 MPa. As can be seen, the film thickness increases as the rotational speed increases from 1 rpm to 2000 rpm, while the contact pressure sharply decreases from 0.25 kPa to 0. The more important thing is that the roughness contact mainly happens on the inner side of the rings, which means there will be a convergent distortion of seal faces.

**Figure 6:**
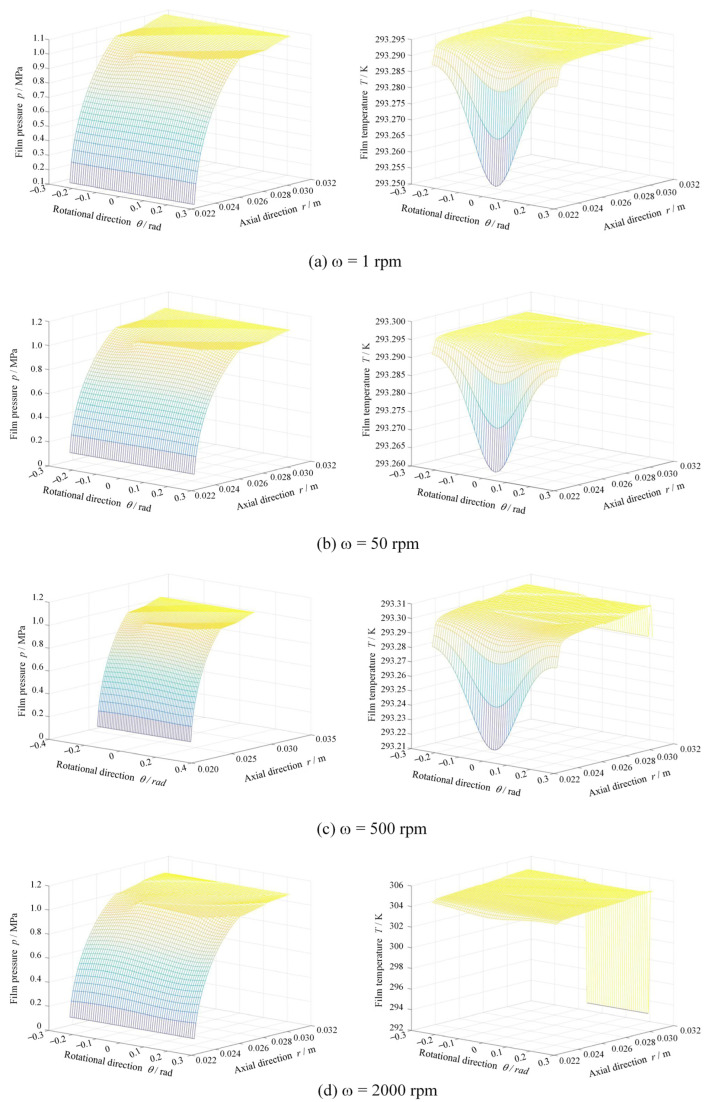
Film pressure and temperature distributions under different rotational speed (*R*a = 0.20 μm, *p*_o_ = 1.1 MPa).

Further, the pressure and temperature distributions of gas film between the seal surfaces can be obtained at different rotational speeds, as shown in Figure 6. Clearly, under low-speed conditions smaller than 500 rpm, there is no significant temperature rise, although there is a contact pressure of 0.25 MPa. It can also be found that the spiral grooves on a sealing surface can produce an obvious hydrodynamic pressure effect due to the function of shear speed when the speed increases to 2000 rpm, while the film temperature increases from 293.3 K to about 306 K.

**Figure 7:**
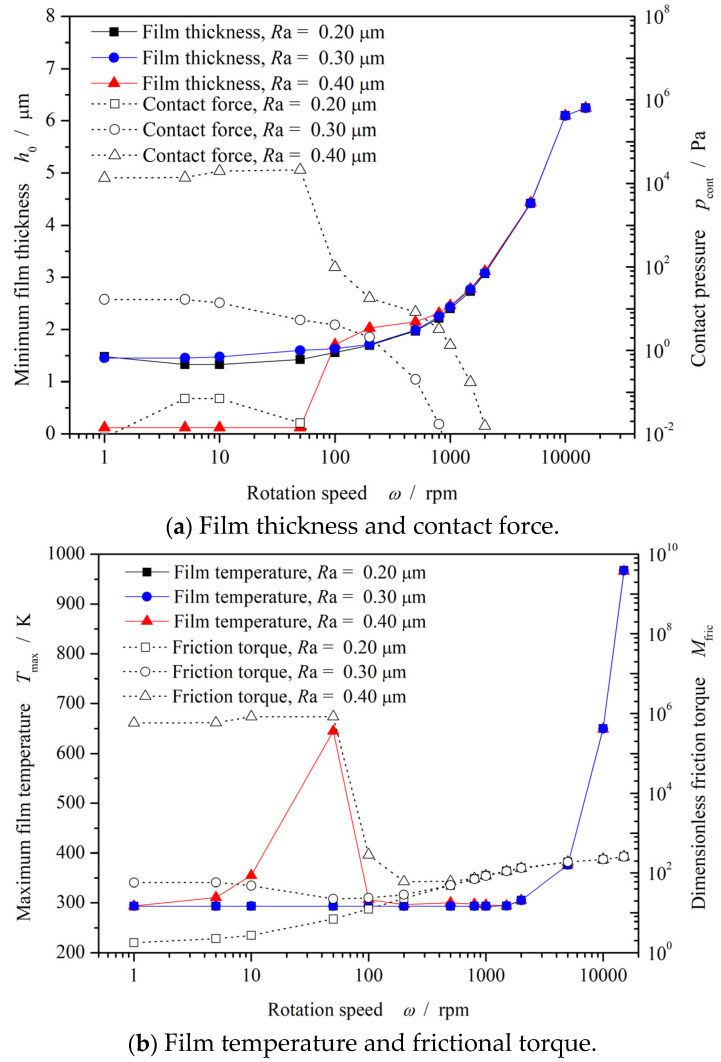
Influence of rotational speed on frictional performance (*p*_o_ = 1.1 MPa).

Figure 7 shows the influence of rotational speed on frictional performance. According to Figure 7a, it can be found that the minimum film thickness demonstrates a tendency to increase with the growth of rotational speed from 0 to 10,000 rpm. Meanwhile, the contact pressure presents a law of first increasing and then decreasing. When the rotational speed increases to a speed exceeding 100 rpm, the value of contact pressure sharply decreases to zero.

As expected in Figure 7b, the frictional torque presents obvious Stribeck curve with increase of rotational speed, especially in the case of *Ra* = 0.4 μm. In another words, the lubrication status transfers from mix-lubrication to full film lubrication when the rotational speed reaches 100 rpm.

Another important conclusion can be found in Figure 7b, which indicates that the greater surface roughness results in a larger temperature rise under low-rotational-speed conditions. For the case of *Ra* = 0.4 μm, the maximum film temperature reaches about 650 K at a rotational speed of 50 rpm. So, the larger surface roughness may result in serious wear at the start-up stage.

### 3.2. Effect of Surface Roughness

It is well known that the surface roughness may lead to a significant temperature rise and risk of surface wear. In particular, the amount of surface roughness is the key factor that directly affects the temperature rise. But it is still not very clear how the magnitude of roughness contact affects the temperature rise. In this part of the paper, the effect of surface roughness on film pressure and temperature is further analyzed.

**Figure 8:**
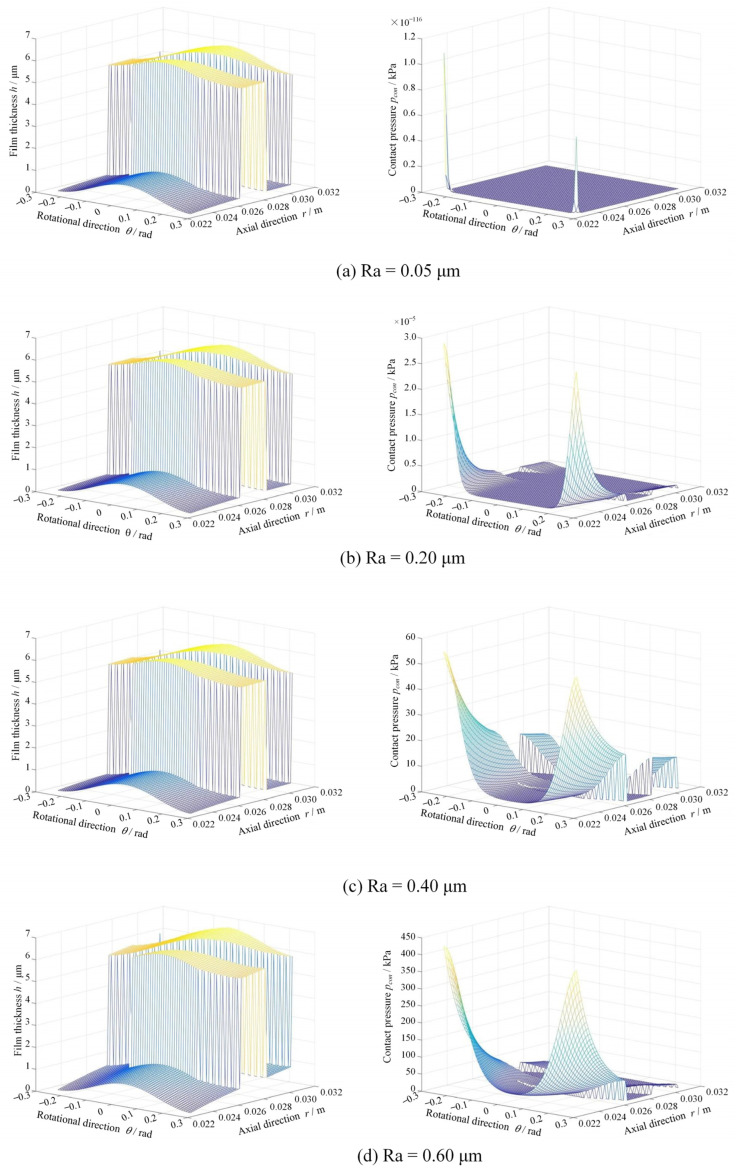
Film thickness and contact pressure distributions at different surface roughness (*ω* = 5000 rpm, *p*_o_ = 1.1 MPa).

**Figure 9:**
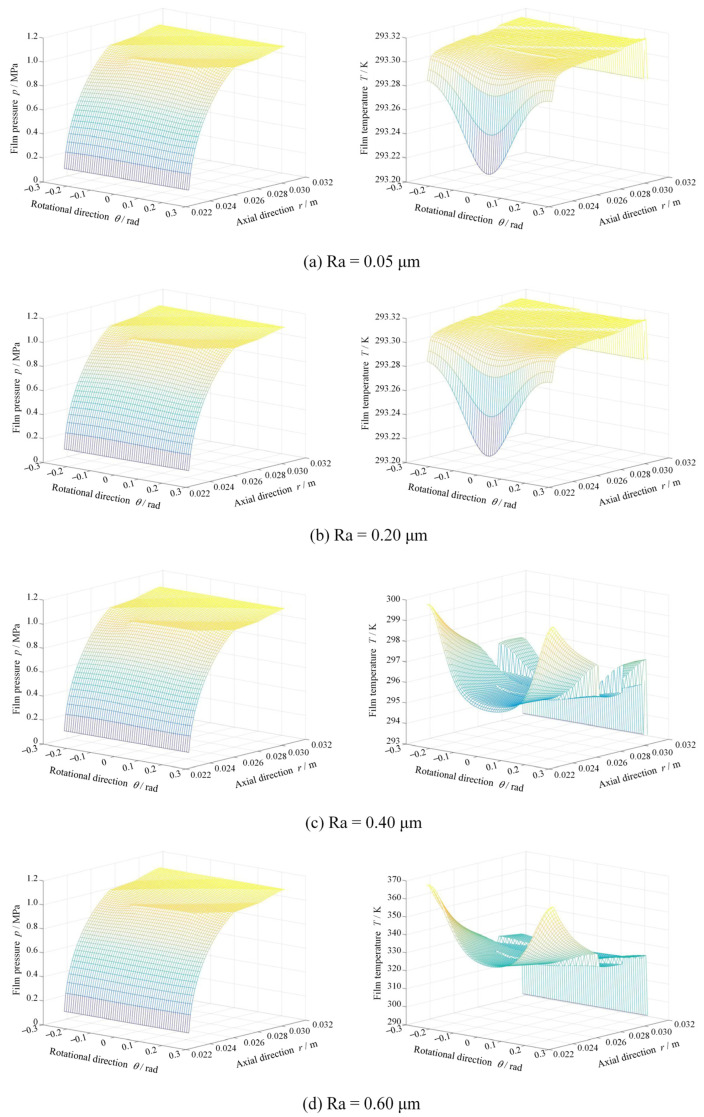
Film pressure and temperature distributions at different surface roughness (*ω* = 5000 rpm, *p*_o_ = 1.1 MPa).

Figure 8 shows the film thickness and contact pressure distributions at different surface roughnesses at *ω* = 5000 rpm and *p*_o_ = 1.1 MPa. Clearly, the contact pressure sharply increases from 0 to 0.4 MPa with the increase in surface roughness from 0.05 μm to 0.60 μm. As a result, there is a significant convergent distortion of about 1 μm in the radius direction for the film thickness, as shown in the figure. The reason is that the increase in surface roughness leads to greater frictional heat, which makes the temperature increase significantly. As shown in Figure 9, when the surface roughness increases from 0.05 μm to 0.60 μm, the maximum film temperature increases from 293.3 K to about 368 K.

**Figure 10:**
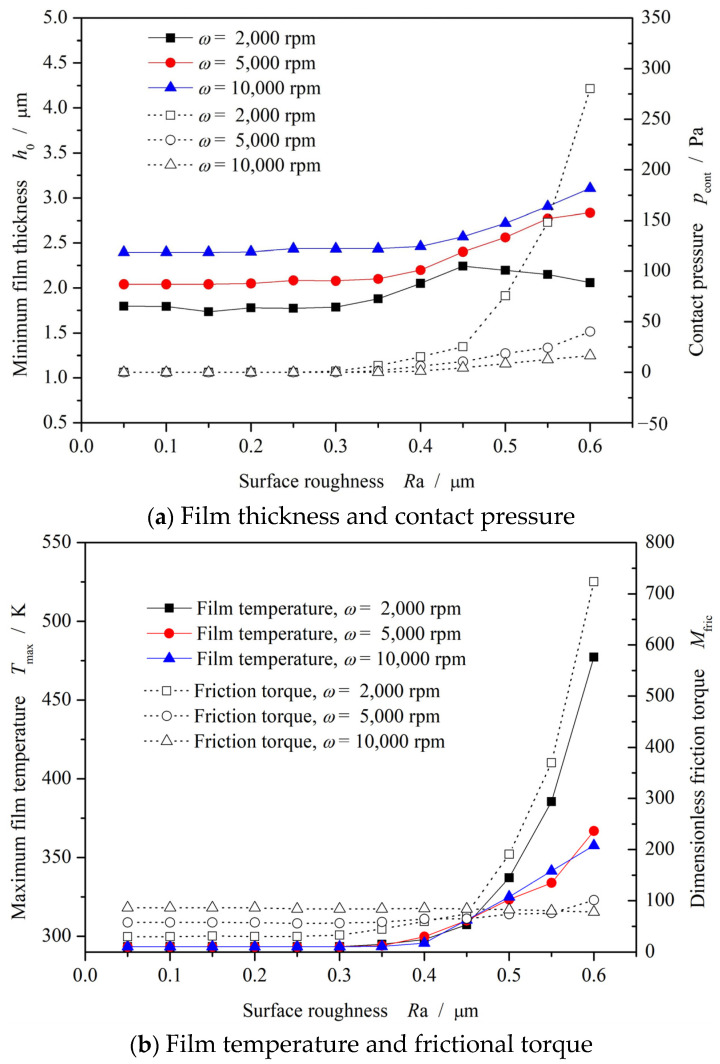
Influence of surface roughness on frictional performance (*p*_o_ = 1.1 MPa).

Figure 10 show the influence of surface roughness on frictional performance. It can be found that, for the spiral groove face seal, the lubricating condition will deteriorate rapidly with increases in the surface roughness after 0.4 μm. Both the contact pressure and frictional torque increase significantly, which leads to a rapid increase in film temperature, exceeding 500 K. This means that excessive roughness increases the risk of wear failure of the seal face.

### 3.3. Effect of Seal Pressure

Seal pressure is another important factor affecting surface contact because of the face distortion. In theory, the greater the seal pressure, the greater the deformation. Due to the uneven deformation of the seal end face, the contact distribution will be very complicated. When designing high-pressure gas face seals, the coupling effect of end deformation and surface contact becomes more important.

**Figure 11:**
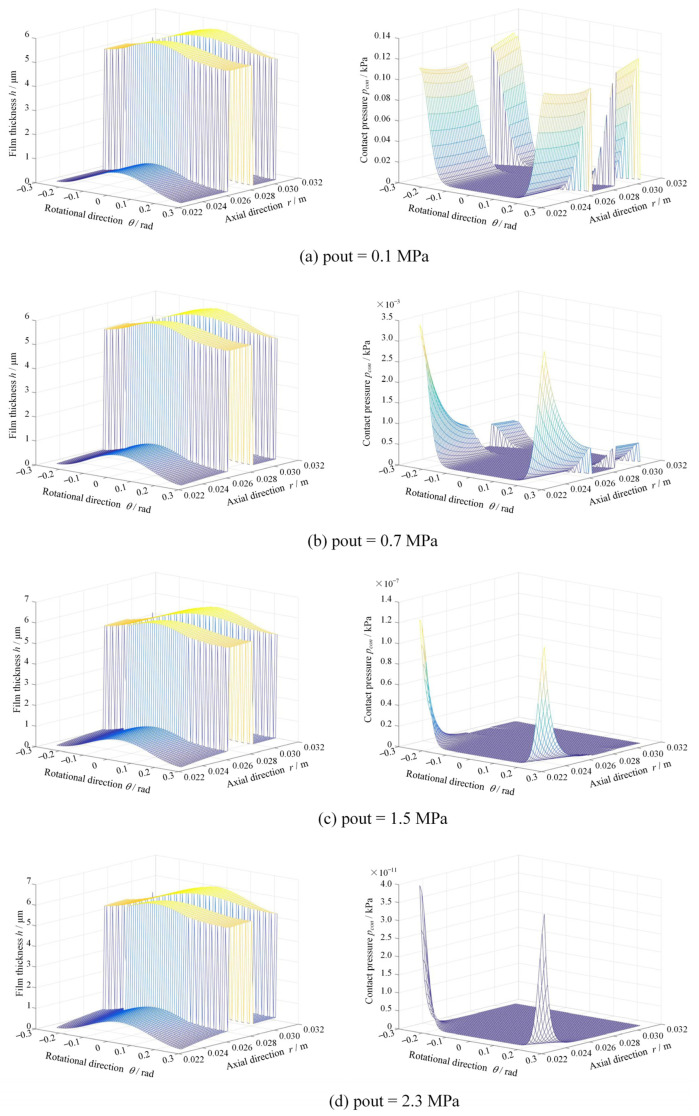
Film thickness and contact pressure distributions at different seal pressure (*R*a = 0.20 μm, *ω* = 5000 rpm).

Figure 11 shows the film thickness and contact pressure distributions at different seal pressures at *R*a = 0.20 μm, *ω* = 5000 rpm. Clearly, the contact pressure sharply decreases from 0.14 kPa to 0 kPa as the seal pressure increases from 0.10 MPa to 2.3 MPa due to the increase in film thickness. The more important result is that, due to smaller face distortion at low seal pressure, the contact pressure presents radial uniform distribution. With the increase in seal pressure, the contact occurs mainly in the inner diameter due to the convergent distortion.

**Figure 12:**
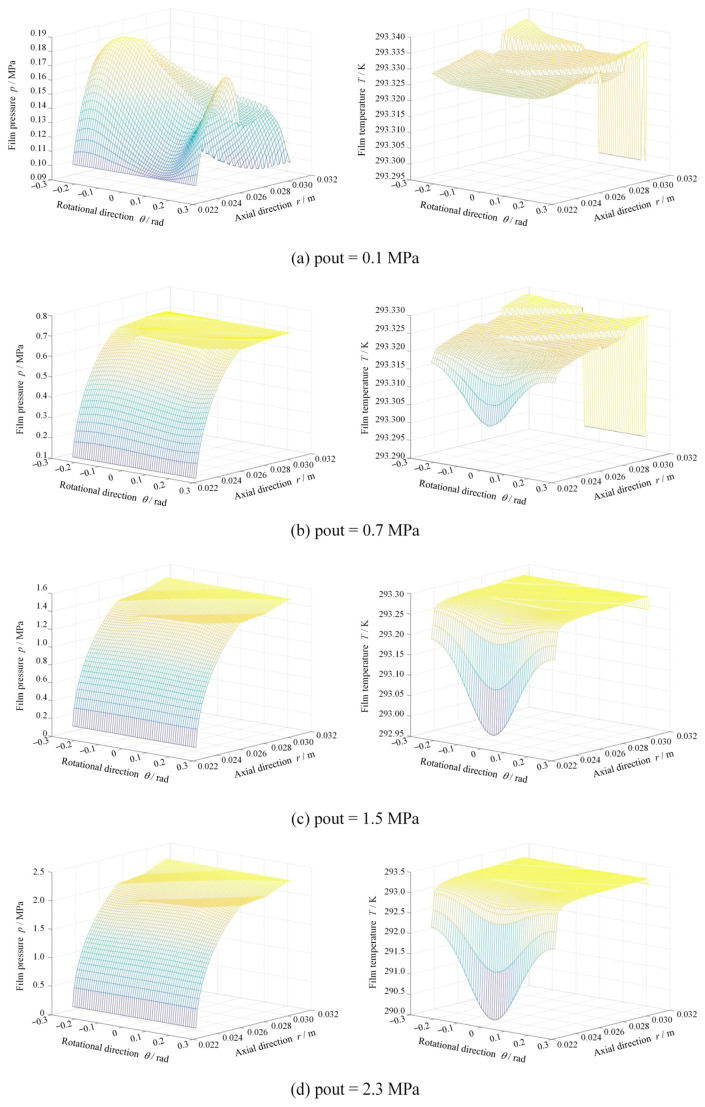
Film pressure and temperature distributions at different seal pressure (*R*a = 0.20 μm, *ω* = 5000 rpm).

Additionally, since there is no obvious contact at higher seal pressure, the film temperature presents no obvious increase, as shown in Figure 12.

**Figure 13:**
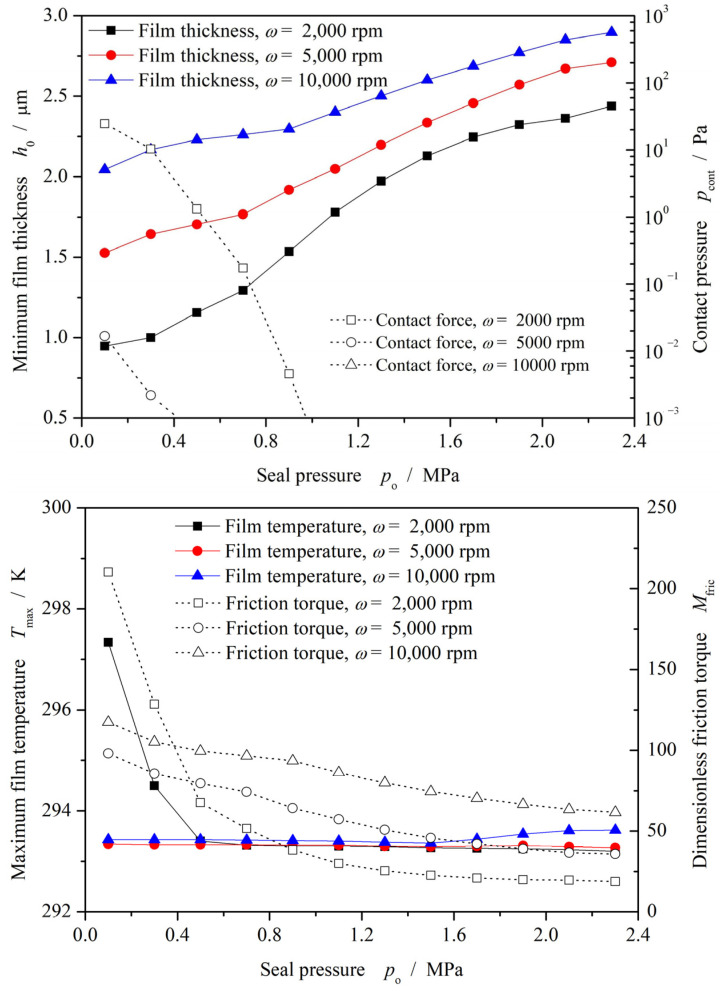
Influence of seal pressure on frictional performance (*R*a = 0.20 μm).

Figure 13 show the influence of seal pressure on frictional performance. It can be found that, for the spiral groove face seal at lower seal pressure, there is an obvious temperature rise due to smaller film thickness and higher contact pressure. With the increase in seal pressure and film thickness, the contact pressure and frictional torque drastically decrease.

## 4. Conclusions

In this paper, based on the elastic contact hypothesis of rough and wavy surfaces and the influence of temperature on the elastic modulus of materials, a thermoelastic contact lubrication model of a gas-lubricated end seal is established. The following conclusions can be drawn.

(a) Surface roughness often leads to significant contact pressure, which mainly happens on the inner side of the rings due to convergent distortion of seal faces, which can easily cause partial wear of the seal faces. Under low-speed conditions smaller than 500 rpm, a contact pressure of 0.25 MPa may be observed.
(b) Greater surface roughness results in a greater temperature rise under low rotational speed and lower seal-pressure conditions, which further increases the risk of severe wear or even failure of the seal faces. In the low-speed cases of 50 rpm, the roughness contact may make the film temperature increase from 293.3 K to 650 K.
(c) The proposed model can not only apply for graphite and steel, but also for other sealing pairs through changing the material parameters. Moreover, the simulated temperature of this model can extend to a larger scale.

## Figures and Tables

**Table 1:** Parameter table of spiral groove seal.

Item and Symbol	Dimensions and Data
Inside radius, *r*_i_	23 mm
Outside radius, *r*_o_	31 mm
Spiral groove radius, *r*_g_	26 mm
Groove numbers, *N*	12
Groove depth, *h*_g_	5 μm
Waviness height, *h*_wave_	0.8 μm
Waviness numbers, *N*_w_	12

**Table 2:** Parameter table of asperity.

Item and Symbol	Dimensions and Data
Topography parameters, *µβσ*	0.075
Standard deviation of asperity height, σ_1_,σ_2_	0.2 µm
Asperity radius, *β*{Tribology: friction and wear of engineering materials}	250 µm
Young’s modulus, *E*_1_	25 GPa
Young’s modulus, *E*_2_	204 GPa

**Table 3:** Characteristics of the seal ring materials.

Characteristics	Graphite	Stainless Steel [25]
Density/kg·m^−3^	1960	7800
Young’s modulus/G·Pa	25	204
Poisson’s coefficient	0.2	0.27
Specific heat capacity/J·kg^−1^·K^−1^	710	460
Thermal conductivity/W·m^−1^·K^−1^	129	16.5
Linear thermal expansion coefficient/10^−6^ °C	4	4.3

## Data Availability

All data is contained with the article.

## Nomenclature

*A*	The actual contact area [mm^2^].
*A*_1_, *A*_2_	Calculation parameters.
*C*	Calculation parameter.
*c*_s__2_	Specific heat capacity [J·kg^−1^·K^−1^].
*c*_v_	The constant specific heat capacity [J·mol^−1^·K^−1^].
*C*_L_	The loss coefficient.
*E*′	Plane-stress modulus [GPa].
*E*, *E*_0_ , *E*_1_ , *E*_2_	Elasticity modulus [GPa].
*E*_m_	Molecular energy per degree of freedom of gas [J·mol^−1^].
*f*	The friction coefficient.
*F*_e_	The cutting force [N].
*h*	Film thickness [m].
*h*_0_	Minimum film clearance [m].
*h*_defrom_	Deflection of film [m].
*h*_g_	Depth of texture [m].
*h*_wave_	The wave amplitude [m].
*i*_d_	Freedom of gas molecules.
*k*_c__1_ , *k*_c__2_	Thermal conductivity of dynamic and static rings, [W·m^−1^·K^−1^].
*k*_gs__1_ , *k*_gs__2_	Heat transfer coefficient [W·m^−2^·K^−1^].
*L*	Measured length [um].
*M*_1_ , *M*_inlet_	The Mach number.
*M*_fric_	Dimensionless friction torque.
*n*	Rotation speed [rpm].
*N*	Groove numbers.
*N*_w_	Waviness numbers.
*p*	Local pressure [Pa].
*p*_o_	Seal pressure [Pa].
*p*_1_ , *p*_2_	Gas pressure [Pa].
*p*_cont_	Contact pressure [Pa].
*p*_i_	Inside pressure [Pa].
*q*	Leakage rate [m^3^·s^−1^].
*Q*	Dimensionless leakage rate.
*Q*_r_	Radial pressure flow factor.
*Q*_s_	Shear flow factor.
*Q* *_θ_*	Circumferential pressure flow factor.
*r*	Radial coordinate [m].
*r*_b_	Balanced radius [m].
*r*_g_	Spiral groove radius [m].
*r*_i_	Inside radius [m].
*r*_o_	Outside radius [m].
*R*	Dimensionless radius.
*Ra*	Contour arithmetic mean [m].
*R*_u_	The universal ideal gas constant [J·mol^−1^·K^−1^].
*T*	Lubrication gas film temperature [K].
*T*_0_	Ambient temperature [K].
*T*_1_	Insider temperature [K].
*T*_2_	Outside temperature [K].
*T*_av_	Average gas temperature [K].
*T*_max_	Maximum film temperature [K].
*T*_s_	Seal torus temperature [K].
*w*	Opening force [N].
*v*, *v*_1_ , *v*_2_	Poisson’s ratio.
*α*, *α*_1_ , *α*_2_ , *α*_3_	Calculation parameters.
*β*	Asperity radius, [m].
*γ*_1_ , *γ*_2_	The expression of parameter.
*η*	Volume viscosity of gas [Pa·s].
*θ*	Rotational direction [rad].
*ω*	Circumferential velocity [rad·s^−1^].
*λ*	The ratio of film thickness to roughness.
*µ* *·* *β* *·* *σ*	Topography parameters.
*ρ*	Local density [mol·m^−3^].
*ρ* *_s_*_2_	Density of rotor ring [kg·m^−3^].
*σ*	The contour root-mean-square deviation [m].
*σ*´	Standard deviation of combined roughness of two Surfaces of σ_1_ and σ_2_ [m].
*σ_s_*	The yield limit of the material [MPa].
